# Cohort profile: PREVENT 1, a nationwide Swedish infant cohort for longitudinal gut microbiome profiling and early-life health outcomes

**DOI:** 10.1136/bmjopen-2026-118233

**Published:** 2026-08-28

**Authors:** Sheila Orwa, Marija Vlajic, Eleonora Cavani, Alfons Edbom Devall, Luisa W Hugerth, Nele Brusselaers, Willem M De Vos

**Affiliations:** 1 Global Health Institute, Department of Family Medicine and Population Health, University of Antwerp, Antwerp, Belgium; 2 Alba Health, Stockholm, Sweden; 3 Department of Medical Biochemistry and Microbiology, Science for Life Laboratory, Uppsala Universitet, Uppsala, Sweden; 4 Department of Women's and Children’s Health, Karolinska Institutet, Stockholm, Sweden; 5 Department of Public Health and Primary Care, Ghent University, Ghent, Belgium; 6 Laboratory of Microbiology, Wageningen University, Wageningen, The Netherlands; 7 Human Microbiome Research Program, University of Helsinki, Helsinki, Finland

**Keywords:** Infant, Gastrointestinal Microbiome, Health, MICROBIOLOGY, PAEDIATRICS, NUTRITION & DIETETICS

## Abstract

**Purpose:**

PREVENT 1 is a nationwide, prospective Swedish infant cohort established to characterise gut microbiome development during the first 2 years of life and to relate microbial trajectories to feeding, infections, growth and everyday well-being. The study integrates repeated infant stool sampling with shotgun metagenomics analysis with aligned parental questionnaires, stool photographs and infant cry recordings collected at three approximately 3-month intervals for each infant.

**Participants:**

Families were recruited nationwide in Sweden from September 2023 through targeted digital channels. Eligible participants were term-born infants residing in Sweden and aged <1 year at enrolment. Baseline questionnaire data and stool samples were collected from 253 infants. Parents completed questionnaires covering socio-demographic characteristics and health, pregnancy and delivery, postnatal factors, infant environment, feeding and growth, infections and other health outcomes, gastrointestinal symptoms and everyday well-being.

**Findings to date:**

Retention was high, with 248 families completing at least one follow-up questionnaire at Phase 2 and 243 at Phase 3. For stool samples, 250 infants provided at least two samples and 241 provided all three. At enrolment, 42.3% of infants were older than 7 months, 73.9% had weight-for-length z-scores in the normal range and exclusive breastfeeding at 4 months was reported for 58.9%.

**Future plans:**

Three-phase sample and questionnaire data collection was completed in December 2024. Future analyses will examine microbiome features, resistome profiles and functional pathways in relation to antibiotic exposure, feeding, growth and infant health outcomes. Subject to ethical approval and participant consent, follow-up may include further stool collection and Swedish register linkage.

**Trial registration number:**

NCT06285630.

**STRENGTHS AND LIMITATIONS OF THIS STUDY**

- The major strength of PREVENT 1 is its nationwide, prospective design with repeated, harmonised data collection across infancy, enabling assessment of within-infant microbiome and symptom trajectories.
- The study combines multimodal phenotyping (stool samples, parent-filled questionnaires, stool photographs and brief cry recordings) collected at prespecified time points.
- Standardised sample stabilisation and central laboratory processing, together with shotgun metagenomics and validated bioinformatics pipelines, support analytical consistency and reproducibility.
- One limitation is reliance on parent-reported outcomes and anthropometry, which may introduce measurement error or misclassification.
- A potential selection toward highly engaged families may affect generalisability.

## Introduction

 The human gut microbiome plays an essential role in orchestrating infant development and long-term health. The first 1000 days from conception represent a critical window in which rapid organ development coincides with the establishment and maturation of the gut microbiome, with lasting implications for allergy, infections, growth, metabolism and neurodevelopment.^1 2^ Additionally, microbiome maturation is a gradual process that continues beyond infancy and into childhood.^3^

Although microbial DNA can be detected in some intrauterine and meconium samples, current evidence suggests that robust resident gut colonisation before birth is unlikely,^4^ and many signals in foetal or first-pass meconium samples appear compatible with low-biomass contamination or very early perinatal acquisition.^5^ Recent conceptual work, therefore, emphasises early-life microbiome transmission in terms of what microbes are acquired, from whom, where and when, with exposures concentrated around birth and early infancy.^6^ From delivery onwards, infant gut communities are highly dynamic and shaped by birth setting, mode of delivery, immediate postnatal care and exposure to intrapartum or early-life antibiotics.^1 7–9^

In breastfed infants, human milk oligosaccharides (HMO) drive enrichment of specialised taxa such as *Bifidobacterium longum* subsp *infantis* and other infant-type bifidobacteria, while introduction of solid foods and increasing environmental contacts are followed by rising richness, evenness and gradual transition towards a more adult-like microbiome.^10 11^ Maturation continues beyond infancy and varies by population, lifestyle, antibiotic exposure, siblings, pets and host genetics.^12–16^ Disruptions from infections, antibiotics or other unfavourable exposures have been linked to later allergic, metabolic, gastrointestinal and neurodevelopmental outcomes.^17 18^

Several longitudinal infant gut microbiome studies have provided important background for the field by describing microbial maturation, key determinants of microbiome development and links with later health outcomes. For example, TEDDY used repeated stool sampling and metagenomic sequencing to characterise gut microbiome development from infancy into early childhood, while HELMi linked repeated infant faecal sampling with extensive questionnaire-based metadata on diet, exposures, lifestyle, health and growth.^19 20^

Despite this progress, important gaps remain in integrating high-resolution metagenomic trajectories with synchronised, home-based measures of everyday infant health and well-being. Parent-observed outcomes such as feeding transitions, intercurrent infections, stooling patterns, gastrointestinal symptoms, sleep disturbance, irritability and crying are often not fully captured in routine healthcare records, although they may reflect day-to-day infant functioning and discomfort. A recent expert review similarly called for broader longitudinal early-life microbiome studies that combine deep microbial profiling with richer contextual phenotyping and more frequent capture of everyday exposures and outcomes.^21^

To address this gap, PREVENT 1 was designed as a prospective Swedish nationwide home-based cohort integrating repeated infant stool metagenomics with synchronised parental questionnaires, stool photographs and brief infant cry recordings. Data were collected across three planned phases, approximately 3 months apart, to chart microbial succession and its associations with feeding, infections, growth and everyday well-being in early infancy.

## Cohort description

### Study overview

PREVENT 1 is a prospective, longitudinal cohort of Swedish infants designed and coordinated by Alba Health, Stockholm. The main aim is to characterise gut microbiome development during the first 2 years of life and link microbial trajectories to feeding, infections, growth and everyday well-being. The study integrates infant stool sampling with aligned parental questionnaires, stool photographs and infant cry recordings, collected at three time points (Phases 1, 2 and 3) scheduled at about 3-month intervals for each infant. All participating parents or legal guardians provided written informed consent before enrolment and could withdraw their child from the study at any time without consequences.

### Eligibility criteria and recruitment

Recruitment began in September 2023. Families were recruited nationwide through targeted advertisement and outreach (primarily via digital channels). Interested families completed an online sign-up form via Typeform indicating basic eligibility. Those meeting inclusion criteria then received detailed study information and consent forms. After written informed consent was returned, Alba Health mailed stool sampling kits and links to the baseline questionnaire.

Eligibility required infants residing in Sweden, born at term (full-term birth as reported by parents), aged under 1 year at enrolment, and at least one parent or legal guardian aged ≥18 years able to complete questionnaires in Swedish or English and to provide written informed consent. Infants on chronic use of systemic antibiotics or proton pump inhibitors at baseline were excluded.

**Figure 1:**
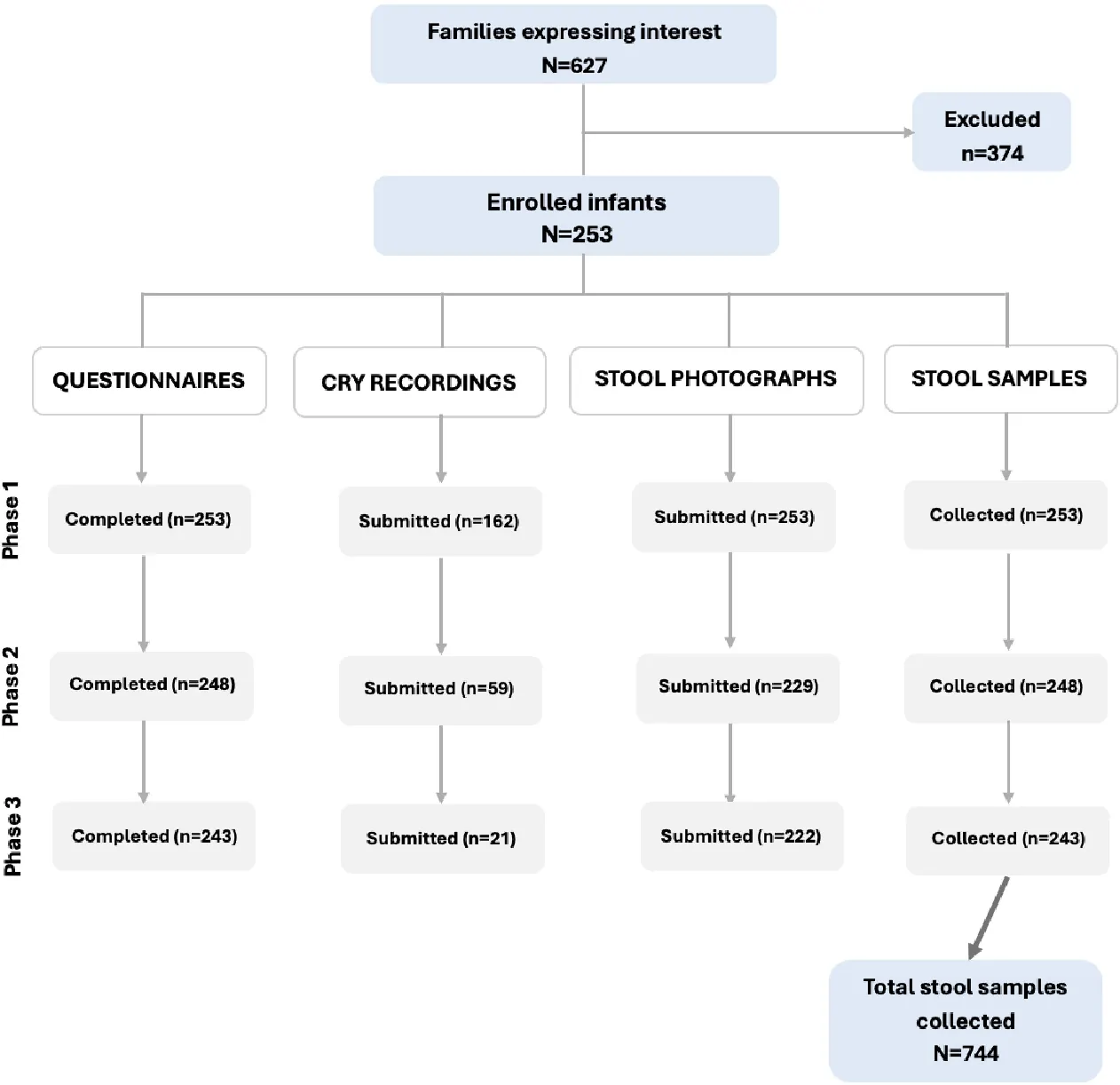
Flowchart showing the number of infants whose parents completed questionnaires and provided stool samples at each phase of data collection of the study. Note: The cry recordings were requested but not mandatory.

Overall, 627 families expressed interest. Of these, 374 did not meet the inclusion criteria or did not complete enrolment procedures. Ultimately, parents of 253 infants completed baseline questionnaires and provided stool samples (Phase 1). Among these, parents of 248 infants completed at least one follow-up questionnaire at Phase 2 and 243 at Phase 3. For stool samples, 250 infants provided at least two stool samples, and 241 provided all three (figure 1; online supplemental efigure 1).

For Phases 2 and 3, families who remained in the study received new stool kits and questionnaire links. Parents could complete stool collection and the questionnaire in either order, provided they were done within 3 days for each phase.

After Phase 3 of the study, families received PDF reports of their third stool sample results and were invited to Q&A sessions with nutritionists and the Alba Health Chief Executive Officer. Post-study surveys captured feedback on behaviour change, child well-being and interest in follow-up. Families who opted in also joined Alba Health’s newsletter and webinars.

### Data collection

The study follows a home-based, parent-led remote sampling design, with families collecting stool samples, digital media (stool photographs and cry recordings) and online questionnaires administered via Typeform. Automated reminders and real-time confirmations of completion were issued to maximise completion.

#### Biological samples

At each phase, parents scooped a small amount of stool directly from the infant’s diaper or, when a diaper was not used, from a potty or toilet using a clean piece of paper and transferred it into a pre-filled tube containing DNA/RNA Shield stabilising buffer (Zymo Research). Tubes were sealed in a provided biosecure mailing pouch and returned via prepaid post at ambient temperature to a central laboratory partner (Zymo Research). On arrival, samples were logged, stored at −80°C and later processed for DNA extraction and metagenomic sequencing.

DNA extraction was performed using the ZymoBIOMICS-96 MagBead DNA Kit following manufacturer protocols. Libraries were prepared with the Illumina DNA Prep (M) Tagmentation kit and sequenced using only Illumina NovaSeq X Plus sequencers, generating paired-end 2×150 base-pair reads with an average depth of ~30 million reads per sample. Sequencing and primary processing are orchestrated using the StaG-mwc workflow.^22^ For this purpose, raw reads underwent quality control with fastp, including adapter trimming and filtering of low-quality reads.^23^ The host DNA was removed with Kraken2 using the human reference genome hg38 as the subtraction database.^24^ Taxonomic profiling was performed with MetaPhlAn V.4.0.6 using a slightly modified version of the mpa_vOct22_CHOCOPhlAnSGB_202212 database, to which two additional strains for *Bifidobacterium longum* were added (*B. longum* subsp *infantis and B. longum* subsp *longum),* according to the Breast Milk Baby cohort.^25^ Functional profiling was conducted with HUMAnN V.3.7 using the default ChocoPhlAn nucleotide database (v_201901_v31) for nucleotide mapping and UniRef90_full for translated searches.^26^

For resistome profiling, quality-controlled and host-depleted shotgun metagenomic reads will be mapped against the ResFinder^27^ database using Bowtie2^28^ to identify antimicrobial resistance genes. Gene counts will be normalised for gene length and sequencing depth and summarised as resistance gene abundance, diversity, presence/absence, antimicrobial class and total antimicrobial resistance gene load. Where relevant, mobile or acquired resistance features captured by the ResFinder-based approach will be considered in exploratory analyses; any additional mobile genetic element-specific workflow will be specified before analysis.

#### Recordings and pictures

Parents were asked to record a single approximately 45 s audio clip of their infant’s crying at each phase. These brief cry recordings will be used for exploratory analyses of infant well-being, including extraction of acoustic features and assessment of their associations with parent-reported excessive crying, irritability and sleep disturbance. Parallel stool photographs were captured to document stool consistency and appearance, which can complement parental reports of gastrointestinal symptoms and facilitate validation of stool form scales. Participation in this component was voluntary and covered by the written informed consent obtained at enrolment.

#### Questionnaires

Parents completed an online questionnaire at matching time points to stool collection, ensuring synchronised capture of environmental and health-related exposures. The first phase required about 60 min, while subsequent phases took about 45 min. Questionnaires were based primarily on the HELMi cohort information.^19^ Relevant items from the HELMi questionnaire were translated and reviewed by the PREVENT 1 research team and collaborators for use in the Swedish study context. The HELMi questionnaire was considered a relevant starting point because it was developed for a Nordic infant cohort with similar early-life microbiome research aims. Items were retained where they captured comparable domains, including infant feeding, medication use, infections, gastrointestinal symptoms, household environment, family lifestyle, health and growth. During early Phase 1 data collection, some wording was refined to improve clarity for participants; however, the underlying constructs and response domains were retained to preserve comparability across phases. Additional PREVENT 1-specific items were included to capture stool photographs, brief infant cry recordings and study-specific follow-up information.

##### Parental demographics and health

Biological parents reported their age at delivery, height and weight. Biological mothers also reported their weight before pregnancy. The parents also provided information on their current and past medical diagnoses, family history of chronic diseases (eg, obesity, diabetes, autoimmune disorders) and lifestyle behaviours, including smoking status and alcohol consumption.

##### Pregnancy and delivery

Mothers reported any previous pregnancies (gravidity, including miscarriages/terminations/ectopics), pregnancy complications (eg, gestational diabetes, pre-eclampsia) and infections during pregnancy. Intrapartum antibiotic use and other medications (eg, analgesics) were self-reported, along with mode of delivery (vaginal delivery, elective caesarean or emergency caesarean). For vaginal births, induction status (induced vs not induced) was captured, but when labour ends in emergency caesarean, prior induction status may be unknown. Exact gestational age, birth weight and Apgar scores were not collected, although only term infants were enrolled.

##### Postnatal factors

Maternal postpartum health was assessed through items on lactation (onset, duration, exclusivity and challenges), physical recovery and psychosocial well-being. Parental stress, sleep and psychosocial well-being were assessed using the following validated Swedish versions of standardised tools: Insomnia Severity Index,^29^ Perceived Stress Scale-10^30^ and Edinburgh Postnatal Depression Scale.^31^ Fathers or co-parents were also asked about their stress and well-being to record family-level context.

##### Infant demographics and environment

Data included infant sex and age at each phase. Parents also reported childcare status (home care vs daycare), recent travel abroad (past 3 months), information on siblings, pets, household composition, housing characteristics (year of construction, ventilation, dampness), rural versus urban residence, presence of a smoker in the household and hygiene practices such as cleaning frequency and exposure to outdoor environments. These variables were included to account for environmental microbial exposures.

##### Infant feeding and growth

Feeding questions captured breastfeeding practices (initiation, ongoing breastfeeding, exclusivity), formula use, combination feeding, use of expressed breast milk or other feeding by bottle and use of supplements such as vitamin D and probiotics. At each phase, parents indicated whether the infant had started solid foods, with options reflecting the level of introduction, for example, ‘taste bites’, ‘food and milk’ and ‘exclusive solid foods’, allowing inference of broad age ranges for solid food introduction. The exact age at which solid food was first introduced was not directly recorded.

Parents provided infant weight and length/height (as measured in child health services or at home), which allows calculation of WHO weight-for-length z-scores (WLZ) using age in days and recumbent length. WLZ categories include severe wasting, wasting, normal, risk of overweight, overweight and obesity.

##### Infant health, gastrointestinal symptoms and well-being

Questionnaires captured common infections such as upper respiratory tract infections, gastroenteritis and otitis media. Hospitalisations or physician visits in the past 3 months, use of any antibiotics, acid-suppressing drugs, gastrointestinal motility and colic medications and any other drugs, such as antipyretics or bronchodilators, were also recorded. Parent-reported gastrointestinal symptoms included episodes of constipation, diarrhoea outside of acute infection, mucus in stool, vomiting, regurgitation, flatulence, infant colic and reflux-like symptoms. Sleep patterns, sleep disturbance, irritability or restlessness, breathing difficulties during feeding, rashes, confirmed eczema diagnosis, wheezing and asthma diagnosis are also recorded as indicators of general well-being and atopic or respiratory morbidity.

### Data analysis

#### Statistical methods

Analyses will exploit the longitudinal design and repeated measures per child. Associations between microbiome features and health outcomes will be analysed using longitudinal mixed-effects models, multivariable regression and selected machine learning approaches. Infant age will be treated as a primary time scale and modelled flexibly to account for maturation patterns. Confounders will be accounted for using directed acyclic graph (DAG)-guided models. Correction for multiple testing will be applied using false discovery rate. In addition, we will link resistome and mobilome profiles, and specific HMO and lactose-degradation pathways, to early-life antibiotic exposure, feeding patterns and growth.

#### Outcomes

Primary outcomes include infant growth trajectories, feeding patterns, infections, allergic manifestations, stool characteristics and well-being (sleep disturbance, irritability/restlessness). Secondary outcomes include gut microbiome composition, diversity and functional pathways (with a particular focus on HMO utilisation, lactose and other carbohydrate metabolism and short-chain fatty acid production) and the gut resistome and mobilome, quantified as the abundance and diversity of antimicrobial resistance genes and associated mobile genetic elements.

#### Confounders

Potential confounders include parental age, pre-pregnancy body mass index (BMI), smoking and alcohol use, chronic diseases, parity, mode of delivery, medication exposure, siblings, pets, household location (urban/suburban/rural) and key infant characteristics such as sex and baseline WLZ. These variables will be selected and structured based on DAGs tailored to each analysis.

### Power analysis

In the ethics submission, it was estimated that a minimum of 100 participants would provide approximately 80% power (α=0.05) to detect between-group differences in overall microbiome composition between groups, corresponding to a standardised mean difference in beta-diversity distances of Δ (delta) ≈ 0.56 (ie, a difference in mean between-group vs within-group distances of ~0.56 SD units), consistent with published microbiome power calculations.^32^ To improve precision for subgroup analyses and longitudinal profiling, the target was set at 250–300 infants with three stool samples and complete questionnaires. With 253 infants currently enrolled and up to three samples per child (744 samples in total), PREVENT 1 is well powered to detect moderate effect sizes in alpha-diversity and key taxa or pathways and to fit adjusted models with approximately 10–15 covariates.

### Representativeness assessment

To assess how representative PREVENT 1 participants were of births in Sweden during the recruitment period, we compared selected maternal baseline characteristics in PREVENT 1 with national aggregate statistics from the Swedish National Board of Health and Welfare for women giving birth in Sweden in 2023 and 2024. Comparisons were limited to characteristics available in the national statistics and measured in a comparable format in PREVENT 1. Characteristics central to PREVENT 1 such as detailed feeding practices and day-to-day gastrointestinal symptoms are not captured in national registry summaries and were therefore not included in the representativeness comparison. We reported counts and percentages and tested differences in distributions using χ^2^ goodness-of-fit tests (table 1).

**Table 1:** Baseline characteristics in the PREVENT 1 cohort compared with all women giving birth in Sweden

	PREVENT 1 cohort (2023–2024) n=253		All pregnant women giving birth in Sweden* (2023-N=94 725): (2024-N=97 779)		P value (χ^2^ goodness-of-fit test)
	n	%	n	%	
**Maternal characteristics**					
Maternal age in years					<0.001
<29	35	13.8	33 462	34.2	
30–34	124	49.0	39 691	40.6	
35–39	74	29.3	19 815	20.3	
>39	20	7.9	4811	4.8	
Maternal pre-pregnancy BMI, in kg/m²					<0.001
<18.5: underweight	13	5.1	2110	2.2	
18.5–24.9: normal weight	157	62.1	49 095	51.8	
25.0–29.9: overweight	58	22.9	26 623	28.1	
>29.9: obese	25	9.9	16 897	17.9	
Any previous pregnancies					0.016
Yes	165	65.2	54 656	57.7	
No	88	34.8	40 069	42.3	
Maternal chronic diseases (any)	74	29.3			
Gestational diabetes	10	3.9	6048	6.38	0.114
Pre-eclampsia	16	6.3	2367	2.5	<0.001
Maternal infection during pregnancy	32	12.6			
Intrapartum antibiotics	60	23.7			
Alcohol use (any)					
Before pregnancy	198	78.3			
During pregnancy	49	19.4			
Smoking (any)					
Before pregnancy	17	6.7	7806	8.4	0.379
During pregnancy	0	0	2590	2.8	0.008
**Paternal characteristics**					
Paternal age in years					
<29	17	6.7			
30–34	99	39.1			
35–39	76	30.1			
>39	45	17.8			
Paternal BMI, in kg/m²					
18.5–24.9: normal weight	125	49.4			
25.0–29.9: overweight	88	34.8			
>29.9: obese	24	9.5			
Missing	16	6.3			
Paternal chronic diseases (any)	50	19.8			
**Infant characteristics**					
Infant age in months					
<4	53	20.9			
4–7	93	36.8			
>7	107	42.3			
Infant sex					0.251
Female	132	52.2	47 815	48.6	
Male	121	47.8	50 636	51.4	
Infant WLZ (WHO)					
Wasting/severe wasting (<−2 SD)	26	10.3			
Normal (≥−2 to ≤+1 SD)	187	73.9			
Risk of overweight (>+1 to ≤+2 SD)	30	11.9			
Overweight/obesity (>+2 SD)	10	4.0			
Delivery mode					0.095
Elective caesarean section	31	12.3	8047	8.5	
Emergency caesarean section	29	11.4	10 653	11.2	
Vaginal	193	76.3	76 025	80.3	
Number of siblings					
0	125	49.4			
1	102	40.3			
>1	26	10.3			
Exclusive breastfeeding					
Fully breastfed, 2 months	184	72.7	68 000	60.9	<0.001
Fully breastfed, 4 months	149	58.9	54 398	49.9	0.004
Fully breastfed, 6 months	53	20.9	13 124	11.9	<0.001
Languages spoken at home					
Swedish alone	156	61.6			
Swedish and other	93	36.8			
No Swedish	4	1.6			

Values are n (%) based on non-missing data. Infant age categories are derived from age in days and presented as <4, 4–7 and >7 months. Chronic conditions include autoimmune disease, asthma, cardiovascular disease, eczema and metabolic syndrome. Swedish reference data are from 2023 for all variables except maternal age and infant sex, which are based on 2024 national statistics. P values are from χ2 goodness-of-fit tests with expected counts derived from Swedish national proportions (2023: n=94 725; 2024: n=97 779) and scaled to the PREVENT 1 sample size (n=253).

Note: ‘No siblings’ reflects the absence of older siblings (previous liveborn children), whereas the maternal characteristics variable captures any prior pregnancy and not any prior live birth.

* Data from the Swedish National Board of Health and Welfare https://www.socialstyrelsen.se/statistik-och-data/statistik/alla-statistikamnen/graviditeter-forlossningar-och-nyfodda/.

BMI, body mass index; WLZ, weight-for-length z-scores.

## Findings to date

### Baseline characteristics

In total, 253 infants were enrolled in the cohort after excluding families who did not meet inclusion criteria or did not complete enrolment.

Baseline characteristics of mothers, fathers and infants are summarised in table 1. Mothers were predominantly 30–34 years old (49.0%). Pre-pregnancy BMI indicated that 62.1% of mothers were of normal weight. Two-thirds of the women had been pregnant before (65.2%). Self-reported intrapartum antibiotics were used in 23.7% of births. Smoking during pregnancy was not reported, while 6.7% of mothers reported smoking before pregnancy, and 19.4% reported alcohol use before they knew that they were pregnant.

At enrolment, all infants were within their first year of life, with 20.9% younger than 4 months, 36.8% aged 4–7 months and 42.3% older than 7 months. WLZ^33^ at baseline indicated that 73.9% were in the normal range, and 4.0% were overweight or obese. Most infants were born vaginally (76.3%), and about half of them (49.4%) had no siblings. Exclusive breastfeeding at 4 months at baseline was reported for 58.9% of infants. Only four infants lived in households where a language other than Swedish was spoken; 61.6% lived in Swedish-only households, and 36.8% lived in households where Swedish was spoken alongside at least one other language.

Compared with all women giving birth in Sweden in 2023 (n=94 725) and 2024 (n=97 779), mothers in the PREVENT 1 cohort tended to be older (78.3% vs 60.9% aged 30–39) and more often had a BMI in the normal range (62.1% vs 51.8%), with lower prevalence of obesity (9.5% vs 17.9%). They were also less likely to smoke during pregnancy (0% vs 2.8%) and slightly more likely to report a history of prior pregnancy (65.2% vs 57.7%). Infant sex distribution aligned closely with national figures (table 1). This comparison is intended to be descriptive, and differences are reported as absolute percentage points for variables available in national aggregate statistics.

### Longitudinal characteristics

Longitudinal exposures and outcomes across the three questionnaire phases are summarised in table 2. At Phase 1 (N=253), Phase 2 (N=248) and Phase 3 (N=243), breastfeeding remained common, with 79.8%, 79.4% and 81.9% of infants, respectively, reported as having received breast milk at least once since birth. Use of formula increased over time (40.3% at Phase 1, 50.8% at Phase 2 and 53.9% at Phase 3). Likewise, the proportion of infants reported as having ever received both breast milk and formula also rose (32.8%, 39.9% and 43.2%). As expected, solid-food consumption increased markedly over phases, with more infants moving from ‘taste bites’ to ‘food and milk’ and ‘exclusive solid foods’ categories by Phase 3.

**Table 2:** Infant characteristics and exposures across the three PREVENT 1 questionnaire phases

Characteristic	Phase 1	Phase 2	Phase 3
	N=253	N=248	N=243
Sex			
Female	132 (52.2)	128 (51.6)	127 (52.3)
Male	121 (47.8)	120 (48.4)	116 (47.7)
Infant WLZ (WHO)			
Wasting/severe wasting (<−2 SD)	26 (10.3)	10 (4.0)	5 (2.1)
Normal (≥−2 to ≤+1 SD*)*	187 (73.9)	167 (67.3)	154 (63.4)
Risk of overweight (>+1 to ≤+2 SD*)*	30 (11.9)	49 (19.8)	59 (24.3)
Overweight/obesity (>+2 SD*)*	10 (4.0)	16 (6.5)	21 (8.6)
Feeding			
Breastfeeding (ever)	202 (79.8)	197 (79.4)	199 (81.9)
Formula feeding (ever)	102 (40.3)	126 (50.8)	131 (53.9)
Formula+breastfeeding	83 (32.8)	99 (39.9)	105 (43.2)
Solid food			
Taste bites	8 (3.2)	34 (13.7)	2 (0.8)
Food and milk	93 (36.8)	148 (59.7)	154 (63.4)
Exclusive solid foods	34 (13.4)	39 (15.7)	85 (35.0)
Vitamin D supplementation	243 (96.0)	225 (90.7)	225 (92.6)
Probiotic supplementation	80 (31.6)	62 (25.0)	39 (16.0)
Medication exposures			
Any antibiotics	11 (4.3)	17 (6.9)	7 (2.9)
Acid suppressants	2 (0.8)	1 (0.4)	0 (0.0)
GI motility and colic medications	23 (9.1)	26 (10.5)	17 (7.0)
Other medication (eg, antipyretics)	59 (23.3)	94 (37.9)	97 (39.9)
Infections and morbidity			
Upper respiratory tract infection	36 (14.2)	42 (16.9)	30 (12.3)
Ear infections (otitis media)	3 (1.2)	7 (2.8)	7 (2.9)
Gastroenteritis/stomach influenza	3 (1.2)	2 (0.8)	2 (0.8)
Skin rash	59 (23.3)	62 (25.0)	57 (23.5)
Skin issues	37 (14.6)	96 (38.7)	90 (37.0)
Eczema diagnosis	21 (8.3)	36 (14.5)	34 (14.0)
Dry cough	13 (5.1)	25 (10.1)	27 (11.1)
Wheezing	34 (13.4)	43 (17.3)	34 (14.0)
Asthma diagnosis	2 (0.8)	6 (2.4)	10 (4.1)
Sought medical care	59 (23.3)	51 (20.6)	27 (11.1)
Gastrointestinal symptoms			
Poop frequency/week			
<7*×*	59 (23.3)	66 (26.6)	39 (16.0)
7×	39 (15.4)	40 (16.1)	40 (16.5)
8–13×	48 (19.0)	69 (27.8)	79 (32.5)
14×	24 (9.5)	30 (12.1)	38 (15.6)
>14*×*	83 (32.8)	37 (14.9)	44 (18.1)
Constipation episode (≥1 episode/week)	12 (4.7)	132 (53.2)	118 (48.6)
Diarrhoea (≥1 episode/week outside infection)	27 (10.7)	58 (23.4)	32 (13.2)
Mucus in stool	50 (19.8)	44 (17.7)	15 (6.2)
Blood in stool	9 (3.6)	7 (2.8)	5 (2.1)
Vomiting (daily)			
1–2×	36 (14.2)	65 (26.2)	22 (9.1)
>2×	64 (25.3)	34 (13.7)	7 (2.9)
Regurgitation (daily)			
1–2×	22 (8.7)	67 (27.0)	33 (13.0)
>2×	48 (19.0)	25 (10.1)	5 (2.1)
Flatulence/excessive gas	83 (32.8)	60 (24.2)	51 (21.0)
Stomach pain	127 (50.2)	78 (31.5)	48 (19.8)
Excessive crying (>3 hours crying/day)	28 (11.1)	33 (13.3)	21 (8.6)
GERD/acid reflux	9 (3.6)	27 (10.9)	10 (4.1)
Well-being and development			
Sleep disturbance/restlessness	55 (21.7)	28 (11.3)	35 (14.4)
Irritability	59 (23.3)	53 (21.4)	46 (18.9)
Breathing difficulties during feeding	10 (4.0)	12 (4.8)	4 (1.6)
Weight gain curve			
Above-average	65 (25.7)	46 (18.5)	39 (16.0)
Normal	149 (58.9)	147 (59.3)	141 (58.0)
Below-average	35 (13.8)	46 (18.5)	55 (22.6)
Childcare and environment			
Daycare attendance	0 (0.0)	1 (0.4)	46 (18.9)
Travel abroad (past 3 months)	22 (8.7)	55 (22.2)	52 (21.4)
Pets in household	78 (30.8)	79 (31.9)	74 (30.5)
Location of the house			
Rural	33 (13.0)	35 (14.1)	33 (13.6)
Suburban	89 (35.2)	90 (36.3)	90 (37.0)
Urban	131 (51.8)	123 (49.6)	120 (49.4)
Smoker in the household	4 (1.6)	4 (1.6)	3 (1.2)

Values are n (%) based on non-missing data for each phase. All variables are parent-reported, except where a physician diagnosis was explicitly reported. NA=missing values. ‘Any antibiotics’ includes any reported systemic antibiotic course during the respective phase. ‘Other medication’ includes other medications such as antipyretics.

GERD, gastro-oesophageal reflux disease; GI, gastrointestinal; WLZ, weight-for-length z-score.

Parent-reported infant antibiotic use per phase was low, ranging from 4.3% to 6.9% and acid-suppressing medication use was rare (two infants at Phase 1 and 1 infant at Phase 2). Use of gastrointestinal motility or colic medications ranged from 9.1% at Phase 1 to 10.5% at Phase 2 and 7.0% at Phase 3, while use of other medications (eg, antipyretics) increased with age (23.3%, 37.9% and 39.9%).

Gastrointestinal symptoms such as constipation, diarrhoea outside acute infection, mucus in stool, vomiting, regurgitation, flatulence, colic and reflux-like symptoms were frequently reported at least once over the follow-up, with varying trajectories by symptom. Across phases, WLZ distributions shifted modestly. The proportion of infants at risk of overweight (>+1 to ≤+2 SD) increased from 11.9% at Phase 1 to 24.3% at Phase 3, while the proportion with severe wasting or wasting decreased. Overweight and obesity remained uncommon (combined ≤8.6% at any phase).

### Characteristics based on age groups

When pooling observations across all three phases (N=744) and grouping them into six age bands (0–3, 4–6, 7–9, 10–12, 13–15 and >15 months) as presented in online supplemental etable 1, most data points fell in the 7–12 months categories, with comparatively fewer observations in early infancy (0–3 months).

**Figure 2:**
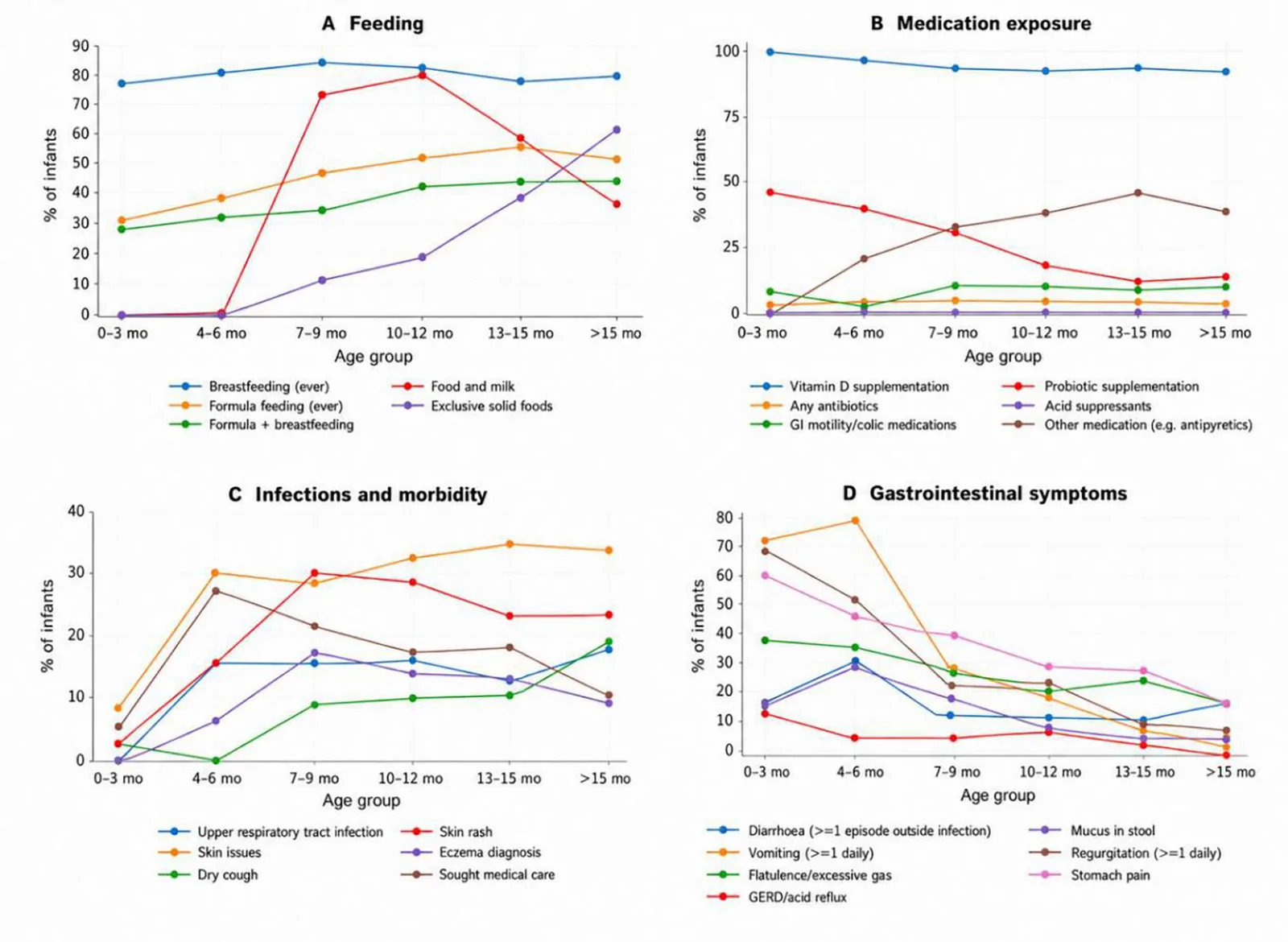
Age-stratified trends in infant characteristics by feeding (A), medication exposure (B), infections and morbidity (C) and gastrointestinal symptoms (D). Line plots show the proportion (%) of infants experiencing various exposures or conditions across six age groups (0–3, 4–6, 7–9, 10–12, 13–15 and >15 months). Note: Percentages reflect parent-reported data; values at each point correspond to the proportion of infants with each characteristic at that age. GERD, gastro-oesophageal reflux disease; GI, gastrointestinal.

The age-stratified table shows expected developmental trends. Feeding patterns shifted from a near-absence of solid foods before 4 months to a rapid rise in ‘food and milk’ from 7 months onward, followed by increasing ‘exclusive solid foods’, from 11.8% at 7–9 months to 41.0% at 13–15 months and 61.5% after 15 months. Skin issues were reported mostly in older infants (figure 2). Similarly, exposures such as travel abroad and daycare attendance appeared almost exclusively in the older age groups (online supplemental etable 1).

**Figure 3:**
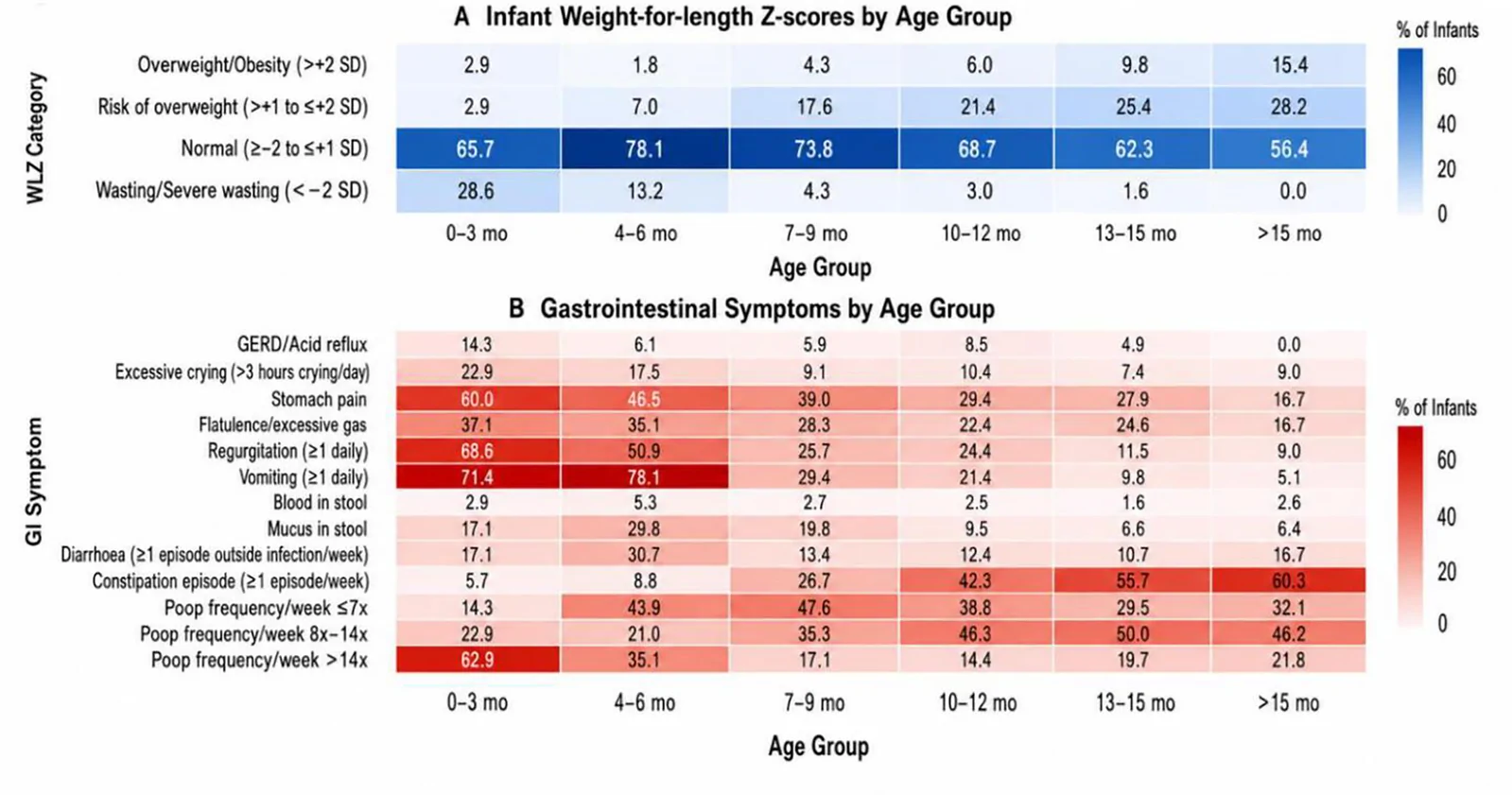
Heatmap visualisation of weight-for-length Z-score (WLZ) status and gastrointestinal symptoms across age groups, expressed in months (mo). (A) Infant WLZ-status based on World Health Organisation (WHO) growth standards. (B) Gastrointestinal symptoms: Including bowel movement frequency and symptoms indicative of gastro-intestinal (GI) distress (eg, reflux, crying, flatulence). Colour intensity reflects the proportion (%) of infants with each characteristic per age group. Darker colours indicate higher prevalence. Percentages are annotated within each cell. GERD, gastro-oesophageal reflux disease.

Markers of undernutrition (severe wasting+wasting) are more common in the youngest age band (about 28% at 0–3 months) and much less frequent beyond 7 months, whereas the proportion at risk of overweight or overweight gradually increases with age. Very frequent stools (>14 times/week) were common (62.9%) in the youngest infants and became less dominant with age, while constipation was reported mostly in older infants (figure 3).

## Strengths and limitations

The strengths of the cohort include its prospective and nationwide design with high retention and the use of repeated, synchronised measurements across infancy, encompassing stool collection, parent questionnaires, stool images and brief cry recordings. Standardised sample stabilisation, central laboratory processing and shotgun metagenomics analysed with validated and widely used bioinformatics pipelines enhance analytical reliability and reproducibility. Notably, the cohort captures sleep disturbance, irritability and gastrointestinal symptoms often missing from registry-based designs, enabling fine-grained analyses of microbiome-well-being relationships over time.

Some noteworthy limitations include reliance on parent-reported outcomes and anthropometry, with the associated risks of measurement error and misclassification, and the absence of maternal microbiome sampling. Another possible limitation is the lack of detailed birth records (exact gestational age, birth weight, Apgar scores, Small (SGA) or large for gestational age (LGA) status). The sample size limits power for rare outcomes and subgroup analyses, and some selection bias toward highly engaged, health-conscious families is possible, which may limit generalisability to populations beyond Swedish infants in similar settings. Because parental contact details are stored in a secure database, families can be re-contacted, subject to appropriate ethical approvals. This enables linkage to national registers and other routinely collected data and supports longer-term follow-up of infant health outcomes and microbiome development.

In conclusion, the PREVENT 1 cohort provides a nationwide, prospective platform with repeated assessments across infancy and standardised sample handling combined with validated metagenomic pipelines. This design supports detailed analyses of symptom patterns and microbiome trajectories, complementing registry-based approaches. Such longitudinal infant microbiome data are timely given growing evidence that early-life *Bifidobacterium* community features and HMO-utilisation capacity are linked to later health outcomes.^34^

### Collaboration

PREVENT 1 metadata will be made available to external investigators following approval of a proposed analysis plan and agreed scope by the principal investigators, and after appropriate data use agreements have been completed. Microbiome and metagenomic data may also be made available for approved collaborative projects, subject to ethical, legal and data protection requirements.

## Supplementary material

10.1136/bmjopen-2026-118233 online supplemental file 1

## Data Availability

Data are available upon reasonable request.
