# Supplementary material for: Cohort profile: PREVENT 1, a nationwide Swedish infant cohort for longitudinal gut microbiome profiling and early-life health outcomes
Source: BMJ Open. 2026 Aug 28;16(8):e118233. doi: 10.1136/bmjopen-2026-118233 (PMC13536151; doi:10.1136/bmjopen-2026-118233)
Supplement: Supplementary file 1 [file bmjopen-2026-118233.reviewer_comments.pdf]

PEER REVIEW HISTORY

BMJ Open publishes all reviews undertaken for accepted manuscripts. Reviewers are asked to complete a checklist review form and are provided with free text boxes to elaborate on their assessment. These free text comments are reproduced below.

ARTICLE DETAILS

Title (Provisional)

Cohort profile: PREVENT 1, a nationwide Swedish infant cohort for longitudinal gut microbiome profiling and early-life health outcomes

Authors

Orwa, Sheila; Vlajic, Marija; Cavani, Eleonora; Devall, Alfons E; Hugerth, Luisa; Brusselaers, Nele; de Vos, Willem

---

VERSION 1 - REVIEW

**Reviewer**	**1**
**Name**	**Taherifard, Ehsan**
**Affiliation**	**Shiraz University of Medical Sciences**
**Date**	**08-Mar-2026**
**COI**	**None**

Dear Authors,

I have reviewed the protocol titled "Longitudinal Gut microbiome profiling and early-life health outcomes in Swedish infants: the PREVENT-1 study." The manuscript is generally well written and represents a valuable study in this field. Below, I provide my comments and suggestions to enhance the quality and clarity of the manuscript.

Major Comments:

1. Some parts of the Introduction are unnecessarily long. Please consider condensing the background information to improve focus. In addition, several longitudinal infant gut microbiome metagenomics studies have already been published. These should be briefly acknowledged, and the Introduction should more clearly specify the gap that those previous studies did not address and how the present study aims to fill it.

2. The manuscript states that the questionnaire was based on the survey used in the HELMi cohort developed for the Finnish population. It would be helpful if the authors could clarify how the questionnaire was adapted for use in the Swedish population. In particular, please indicate whether a formal translation and cultural adaptation process was undertaken (e.g., forward–back translation, expert review, pilot testing), and whether any items were

modified or additional items were introduced. For transparency and reproducibility, it would also be useful to provide the full questionnaire as supplementary material. If the questionnaire is available only in Swedish, an English translation would be helpful for readers.

3. The methods section describes stool collection, sequencing, and taxonomic/functional profiling. However, the assessment of secondary outcomes such as the resistome and mobilome is not described.

4. Comparing the sample counts directly with large population counts using a Pearson chi-square test may lead to exaggerated statistical significance. A more appropriate approach would be a chi-square goodness-of-fit test, in which the expected counts are derived from the known population proportions and scaled to the sample size. This method evaluates whether the observed sample distribution differs from the population distribution without inflating significance due to population size.

Minor Comments:

1. The manuscript states that the postnatal factors section of the questionnaire includes the ISI scale, the PSS-10 scale, and the EPDS. Please clarify whether validated Swedish versions were used, and if so, provide the relevant references.

2. I assume that the WLZ categories ranging from severe wasting to obesity are based on the WHO Child Growth Standards. Please provide the appropriate citation to support this classification.

**Reviewer**	**2**
**Name**	**Reimer, Raylene A.**
**Affiliation**	**University of Calgary, Faculty of Kinesiology**
**Date**	**08-Jun-2026**
**COI**	**None**

The manuscript by Orwa et al. describes the PREVENT-1 longitudinal cohort of Swedish infants. The study focuses on the important relationship between the gut microbiome and early-life health outcomes. The manuscript describes the characteristics of the cohort and its' strengths and limitations. The manuscript is well-written and there are just a few considerations to improve the paper.

Comments:

1. The wording of the purpose in the Abstract is awkward. I would suggest: The study integrates repeated infant stool sampling with shotgun metagenomics analysis with aligned parental questionnaires, stool photographs, and infant cry recordings collected at three approximate 3-month intervals for each infant.

2. Did the authors collect information on whether breastfeeding was feeding at the breast or pumped and given in a bottle? This could be included under the Infant feeding and growth section. It may have an influence on the gut microbiome.

3. In the Infant feeding and growth section of the Cohort Description, it says that "At each phase, parents indicated whether the infant had started solid foods", however, in Table 2 there is no data presented for Phase I where it says NA. Given that infants were enrolled at any age less than 1 year, there would be some infants consuming solid foods. Can this data be added to Table 2?

4. Can the authors indicate what will be done with the crying recordings.

5. The font size is small and difficult to read in Figure 2.

VERSION 1 - AUTHOR RESPONSE

Reviewer 1
Major comment 1
"Some parts of the Introduction are unnecessarily long. Please consider condensing the background information to improve focus. In addition, several longitudinal infant gut microbiome metagenomics studies have already been published. These should be briefly acknowledged, and the Introduction should more clearly specify the gap that those previous studies did not address and how the present study aims to fill it."
Response:
We have shortened and refocused the Introduction to reduce broad background detail and improve clarity. We now briefly acknowledge previous longitudinal infant gut microbiome studies, including TEDDY and HELMi, and explain that these studies have provided important background by describing microbial maturation, determinants of microbiome development and links with later health outcomes. We have also clarified the specific gap addressed by PREVENT 1: the integration of high-resolution metagenomic trajectories with synchronised, home-based measures of everyday infant health and well-being captured close to stool sampling.
Major comment 2
"The manuscript states that the questionnaire was based on the survey used in the HELMi cohort developed for the Finnish population. It would be helpful if the authors could clarify how the questionnaire was adapted for use in the Swedish population. In particular, please indicate whether a formal translation and cultural adaptation process was undertaken (e.g., forward–back translation, expert review, pilot testing), and whether any items were modified or additional items were introduced. For transparency and reproducibility, it would also be useful to provide the full questionnaire as supplementary material. If the questionnaire is available only in Swedish, an English translation would be helpful for readers."
Response:

We have expanded the Questionnaire section to clarify how the questionnaire was adapted for PREVENT 1. Relevant items from the HELMi questionnaire were translated and reviewed by the PREVENT 1 research team and collaborators for use in the Swedish study context. The HELMi questionnaire was considered a relevant starting point because it was developed for a Nordic infant cohort with similar early-life microbiome research aims. Items were retained where they captured comparable domains. We also clarified that some wording was refined during early Phase 1 data collection to improve participant clarity, while retaining the same underlying constructs and response domains to preserve comparability across phases. Additional PREVENT 1-specific items were introduced to capture stool photographs, brief infant cry recordings and study-specific follow-up information. No separate formal forward–back translation, cultural validation or pilot validation study was conducted. We have added more detailed information on the questionnaire origin, adaptation process and content domains to improve transparency and reproducibility.

Major comment 3

"The methods section describes stool collection, sequencing, and taxonomic/functional profiling. However, the assessment of secondary outcomes such as the resistome and mobilome is not described."

Response:

We have added a description of the planned resistome profiling approach in the Biological samples section. The revised manuscript now states that quality-controlled and host-depleted shotgun metagenomic reads will be mapped against the ResFinder database using Bowtie2 to identify antimicrobial resistance genes. We have also clarified that mobile or acquired resistance features captured by the ResFinder-based approach will be considered in exploratory analyses, and that any additional mobile genetic element-specific workflow will be specified before analysis.

Major comment 4

"Comparing the sample counts directly with large population counts using a Pearson chi-square test may lead to exaggerated statistical significance. A more appropriate approach would be a chi-square goodness-of-fit test, in which the expected counts are derived from the known population proportions and scaled to the sample size. This method evaluates whether the observed sample distribution differs from the population distribution without inflating significance due to population size."

Response:

We agree with the reviewer and have revised the representativeness assessment accordingly. We now use chi-square goodness-of-fit tests, with expected counts derived from Swedish national proportions and scaled to the PREVENT 1 sample size. The Methods section and Table 1 heading and footnote have been revised to clarify this approach.

Minor comment 1

"The manuscript states that the postnatal factors section of the questionnaire includes the ISI scale, the PSS-10 scale, and the EPDS.

Please clarify whether validated Swedish versions were used, and if so, provide the relevant references."
Response:
We have clarified in the Postnatal factors section that validated Swedish versions of the Insomnia Severity Index, Perceived Stress Scale-10 and Edinburgh Postnatal Depression Scale were used. We have also added the relevant validation references.
Minor comment 2
"I assume that the WLZ categories ranging from severe wasting to obesity are based on the WHO Child Growth Standards. Please provide the appropriate citation to support this classification."
Response:
We have clarified that infant weight-for-length z-score categories were based on the World Health Organization Child Growth Standards. We have added the relevant WHO citation.

Reviewer 2
Comment 1
"The wording of the purpose in the Abstract is awkward. I would suggest: The study integrates repeated infant stool sampling with shotgun metagenomics analysis with aligned parental questionnaires, stool photographs, and infant cry recordings collected at three approximate 3-month intervals for each infant."
Response:
We have revised the Purpose section of the Abstract to improve clarity and have incorporated the suggested wording.
Comment 2
"Did the authors collect information on whether breastfeeding was feeding at the breast or pumped and given in a bottle? This could be included under the Infant feeding and growth section. It may have an influence on the gut microbiome."
Response:
We collected information on feeding using a bottle, including expressed breast milk or other bottle feeding, and have clarified this in the Infant feeding and growth section.
Comment 3
"In the Infant feeding and growth section of the Cohort Description, it says that "At each phase, parents indicated whether the infant had started solid foods"; however, in Table 2 there is no data presented for Phase I where it says NA. Given that infants were enrolled at any age less than 1 year, there would be some infants consuming solid foods. Can this data be added to Table 2?"
Response:
We have revised Table 2 to include solid-food data for Phase 1.
Comment 4
"Can the authors indicate what will be done with the crying recordings?"
Response:

We have added clarification in the Recordings and pictures section. The revised manuscript states that the brief infant cry recordings will be used for exploratory analyses of infant well-being, including extraction of acoustic features and assessment of their associations with parent-reported excessive crying, irritability and sleep disturbance.
Comment 5
“The font size is small and difficult to read in Figure 2.”
Response:
We have revised Figure 2 to improve readability. We also updated the figure to reflect the corrected age-stratified feeding values.

VERSION 2 - REVIEW

**Reviewer**	**1**
**Name**	**Taherifard, Ehsan**
**Affiliation**	**Shiraz University of Medical Sciences**
**Date**	**27-Jul-2026**
**COI**	

Dear Authors,

I have reviewed the revised version of protocol titled " Cohort profile: PREVENT 1, a nationwide Swedish infant cohort for longitudinal gut microbiome profiling and early-life health outcomes." I appreciate the authors’ efforts in addressing my previous comments.

Comments:

1. Thank you for clarifying the questionnaire adaptation process. However, I could not identify the full questionnaire (or an English translation of the Swedish version) among the supplementary materials.

**Reviewer**	**2**
**Name**	**Reimer, Raylene A.**
**Affiliation**	**University of Calgary, Faculty of Kinesiology**
**Date**	**08-Jul-2026**
**COI**	

No further comments.
