# Supplementary material for: Cohort profile: PREVENT 1, a nationwide Swedish infant cohort for longitudinal gut microbiome profiling and early-life health outcomes
Source: BMJ Open. 2026 Aug 28;16(8):e118233. doi: 10.1136/bmjopen-2026-118233 (PMC13536151; doi:10.1136/bmjopen-2026-118233)
Supplement: Supplementary file 2 [file bmjopen-16-8-s001.docx]

**SUPPLEMENTARY MATERIAL**

**Cohort profile: PREVENT 1, a nationwide Swedish infant cohort for longitudinal gut microbiome profiling and early-life health outcomes**

eFigure 1: Distribution of infant age at stool sample collection across PREVENT 1 phases**.**

eTable 1: Infant characteristics and exposures by age band across all PREVENT 1 observations.

**eFigure 1: Distribution of infant age at stool sample collection across PREVENT 1 phases.**
Stacked bars show the number of samples in each study phase (1-3), coloured by infant age band at the time of sampling (0-3, 4-6, 7-9, 10-12, 13-15 and >15 months). “Missing” indicates samples with missing age information.

**eTable 1: Infant characteristics and exposures by age band across all PREVENT 1 observations**

| **Characteristics** | **0 - 3 months** | **4 - 6 months** | **7 - 9 months** | **10 - 12 months** | **13-15 months** | **>15 months** |
| --- | --- | --- | --- | --- | --- | --- |
|  | **N=35** | **N=114** | **N=187** | **N=201** | **N=122** | **N=78** |
| **Sex** |  |  |  |  |  |  |
| Female | 20 (57.1) | 48 (42.1) | 93 (49.7) | 107 (53.2) | 63 (51.6) | 54 (68.2) |
| Male | 15 (42.9) | 66 (57.9) | 94 (50.3) | 94 (46.8) | 59 (48.4) | 24 (30.8) |
| Infant WLZ (WHO) |  |  |  |  |  |  |
| *Wasting /Severe wasting (<-2 SD)* | 10 (28.6) | 15 (13.2) | 8 (4.3) | 6 (3.0) | 2 (1.6) | 0 (0.0) |
| *Normal (≥-2 to ≤+1 SD)* | 23 (65.7) | 89 (78.1) | 138 (73.8) | 138 (68.7) | 76 (62.3) | 44 (56.4) |
| *Risk of overweight (>+1 to ≤+2 SD)* | 1 (2.9) | 8 (7.0) | 33 (17.6) | 43 (21.4) | 31 (25.4) | 22 (28.2) |
| *Overweight/Obesity (>+2 SD)* | 1 (2.9) | 2 (1.8) | 8 (4.3) | 12 (6.0) | 12 (9.8) | 12 (15.4) |
| **Feeding** |  |  |  |  |  |  |
| Breastfeeding (ever) | 27 (77.1) | 92 (80.7) | 157 (84.0) | 165 (82.1) | 95 (77.9) | 62 (79.5) |
| Formula feeding (ever) | 11 (31.4) | 44 (38.6) | 88 (47.1) | 106 (52.7) | 69 (56.6) | 41 (52.6) |
| Formula + breastfeeding | 10 (28.6) | 37 (32.5) | 65 (34.8) | 86 (42.8) | 54 (44.3) | 35 (44.9) |
| Solid food |  |  |  |  |  |  |
| *Taste bites* | 0 (0.0) | 23 (20.2) | 20 (10.7) | 1 (0.5) | 0 (0.0) | 0 (0.0) |
| *Food and milk* | 0 (0.0) | 1 (0.9) | 140 (74.9) | 164 (81.6) | 71 (58.2) | 29 (37.2) |
| *Exclusive solid foods* | 0 (0.0) | 0 (0.0) | 22 (11.8) | 39 (19.4) | 50 (41.0) | 48 (61.5) |
| Vitamin D supplementation | 35 (100) | 110 (96.5) | 175 (93.6) | 187 (93.0) | 114 (93.4) | 72 (92.3) |
| Probiotic supplementation | 16 (45.7) | 45 (39.5) | 57 (30.5) | 37 (18.4) | 15 (12.3) | 11 (14.1) |
| **Medication exposures** |  |  |  |  |  |  |
| Any antibiotics | 1 (2.9) | 5 (4.4) | 10 (5.3) | 10 (5.0) | 6 (4.9) | 3 (3.8) |
| Acid suppressants | 0 (0.0) | 1 (0.9) | 1 (0.5) | 1 (0.5) | 0 (0.0) | 0 (0.0) |
| GI motility and colic medications | 3 (8.6) | 3 (2.6) | 20 (10.7) | 21 (10.4) | 11 (9.0) | 8 (10.3) |
| Other medication (e.g. antipyretics) | 0 (0.0) | 24 (21.1) | 63 (33.7) | 77 (38.3) | 56 (45.9) | 30 (38.5) |
| **Infections and morbidity** |  |  |  |  |  |  |
| Upper respiratory tract infection | 0 (0.0) | 18 (15.8) | 29 (15.5) | 32 (15.9) | 15 (12.3) | 14 (17.9) |
| Ear infections (otitis media) | 0 (0.0) | 1 (0.9) | 2 (1.1) | 7 (3.5) | 4 (3.3) | 3 (3.8) |
| Gastroenteritis/stomach flu | 0 (0.0) | 0 (0.0) | 4 (2.1) | 2 (1.0) | 0 (0.0) | 1 (1.3) |
| Skin rash | 1 (2.9) | 18 (15.8) | 56 (29.9) | 57 (28.4) | 28 (23.0) | 18 (23.1) |
| Skin issues | 3 (8.6) | 34 (29.8) | 53 (28.3) | 65 (32.3) | 42 (34.4) | 26 (33.3) |
| Eczema diagnosis | 0 (0.0) | 7 (6.1) | 33 (17.6) | 28 (13.9) | 16 (13.1) | 7 (9.0) |
| Dry cough | 1 (2.9) | 0 (0.0) | 17 (9.1) | 20 (10.0) | 13 (10.7) | 15 (19.2) |
| Wheezing | 0 (0.0) | 4 (3.5) | 35 (18.7) | 40 (19.9) | 18 (14.8) | 14 (17.9) |
| Asthma diagnosis | 0 (0.0) | 0 (0.0) | 3 (1.6) | 7 (3.5) | 3 (2.5) | 5 (6.4) |
| Sought medical care | 2 (5.7) | 31 (27.2) | 39 (20.9) | 35 (17.4) | 22 (18.0) | 8 (10.3) |
| **Gastrointestinal symptoms** | |  |  |  |  |  |
| Poop frequency/week |  |  |  |  |  |  |
| *≤7x* | 5 (14.3) | 50 (43.9) | 89 (47.6) | 78 (38.8) | 36 (29.5) | 25 (32.1) |
| *8x-14x* | 8 (22.9) | 24 (21.0) | 66 (35.3) | 93 (46.3) | 61 (50.0) | 36 (46.2) |
| *>14x* | 22 (62.9) | 40 (35.1) | 32 (17.1) | 29 (14.4) | 24 (19.7) | 17 (21.8) |
| Constipation episode (≥1 episode/week) | 2 (5.7) | 10 (8.8) | 50 (26.7) | 85 (42.3) | 68 (55.7) | 47 (60.3) |
| Diarrhoea (≥1 episode outside infection/week) | 6 (17.1) | 35 (30.7) | 25 (13.4) | 25 (12.4) | 13 (10.7) | 13 (16.7) |
| Mucus in stool | 6 (17.1) | 34 (29.8) | 37 (19.8) | 19 (9.5) | 8 (6.6) | 5 (6.4) |
| Blood in stool | 1 (2.9) | 6 (5.3) | 5 (2.7) | 5 (2.5) | 2 (1.6) | 2 (2.6) |
| Vomiting (≥1 daily) | 25 (71.4) | 89 (78.1) | 55 (29.4) | 43 (21.4) | 12 (9.8) | 4 (5.1) |
| Regurgitation (≥1 daily) | 24 (68.6) | 58 (50.9) | 48 (25.7) | 49 (24.4) | 14 (11.5) | 7 (9.0) |
| Flatulence/excessive gas | 13 (37.1) | 40 (35.1) | 53 (28.3) | 45 (22.4) | 30 (24.6) | 13 (16.7) |
| Stomach pain | 21 (60.0) | 53 (46.5) | 73 (39.0) | 59 (29.4) | 34 (27.9) | 13 (16.7) |
| Excessive crying (>3 hours crying/day) | 8 (22.9) | 20 (17.5) | 17 (9.1) | 21 (10.4) | 9 (7.4) | 7 (9.0) |
| GERD/Acid reflux | 5 (14.3) | 7 (6.1) | 11 (5.9) | 17 (8.5) | 6 (4.9) | 0 (0.0) |
| **Wellbeing and development** |  |  |  |  |  |  |
| Sleep disturbance/restlessness | 8 (22.9) | 19 (16.7) | 27 (14.4) | 29 (14.4) | 21 (17.4) | 13 (16.7) |
| Irritability | 9 (25.7) | 29 (25.4) | 37 (19.8) | 43 (21.4) | 25 (20.5) | 15 (19.2) |
| Breathing difficulties during feeding | 4 (11.4) | 10 (8.8) | 2 (1.1) | 6 (3.0) | 3 (2.5) | 1 (1.3) |
| Weight gain curve |  |  |  |  |  |  |
| *Above-average* | 8 (22.9) | 37 (32.5) | 38 (20.3) | 39 (19.4) | 16 (13.1) | 12 (15.4) |
| *Normal* | 21 (60.0) | 55 (48.2) | 115 (61.5) | 121 (60.2) | 74 (60.7) | 51 (65.4) |
| *Below-average* | 5 (14.3) | 22 (19.3) | 32 (17.1) | 38 (18.9) | 29 (23.8) | 10 (12.8) |
| **Childcare and environment** |  |  |  |  |  |  |
| Daycare attendance | 0 (0.0) | 0 (0.0) | 0 (0.0) | 0 (0.0) | 11 (9.0) | 36 (46.2) |
| Travel abroad (past 3 months) | 0 (0.0) | 17 (14.9) | 37 (19.8) | 42 (20.9) | 23 (18.9) | 10 (12.8) |
| Pets in household | 11 (31.4) | 33 (28.9) | 62 (33.2) | 63 (31.3) | 40 (32.8) | 23 (29.5) |
| Location of the house |  |  |  |  |  |  |
| *Rural* | 4 (11.4) | 14 (12.3) | 26 (22.8) | 27 (13.4) | 18 (14.8) | 12 (15.4) |
| *suburban* | 10 (28.6) | 37 (32.5) | 72 (38.5) | 73 (36.3) | 48 (39.3) | 25 (32.1) |
| *Urban* | 21 (60) | 63 (55.3) | 89 (47.6) | 101 (50.3) | 56 (45.9) | 41 (52.6) |
| Smoker in the household | 0 (0.0) | 2 (1.8) | 3 (1.6) | 5 (2.5) | 1 (0.8) | 1 (1.3) |

Values are n (%) based on non-missing data for each age group; N refers to the number of questionnaire observations (across all phases), not unique infants. Infant age categories are derived from age in days and presented as 0-3, 4-6, 7-9, 10-12, 13-15 and >15 months. All variables are parent-reported, except where a physician diagnosis was explicitly reported. WLZ (WHO) = World Health Organization weight-for-length z-score. NA = missing values. “Any antibiotics” includes any reported systemic antibiotic course during the respective age interval. “Other medication” includes other medications such as antipyretics.
